# Interpretation of Frequency Effect for High-Strength Steels with Three Different Strength Levels via Crystal Plasticity Finite Element Method

**DOI:** 10.3390/ma17102350

**Published:** 2024-05-15

**Authors:** Yingxin Zhao, Xiaoya Wang, Like Pan, Jun Wang, Liming Chen, Tong Xing, Junchen Zhu, Aiguo Zhao

**Affiliations:** 1 Standards & Metrology Research Institute, China Academy of Railway Sciences Corporation Limited, Beijing 100010, China; yxzhao15801042226@163.com (Y.Z.); 15811255420@163.com (X.W.); 13810305638@163.com (L.C.); xingtong2001@126.com (T.X.); 2 State Key Laboratory of Nonlinear Mechanics, Institute of Mechanics, Chinese Academy of Sciences, Beijing 100190, China; wangjun@lnm.imech.ac.cn; 3 College of Civil Engineering, Nanjing Tech University, Nanjing 211816, China; zhujc980216@foxmail.com (J.Z.); shiang_37@163.com (A.Z.)

**Keywords:** frequency effect, very-high-cycle fatigue, crystal plasticity finite element method, plastic strain accumulation, fatigue life prediction

## Abstract

The fatigue behavior of a high-strength bearing steel tempered under three different temperatures was investigated with ultrasonic frequency and conventional frequency loading. Three kinds of specimens with various yield strengths exhibited obvious higher fatigue strengths under ultrasonic frequency loading. Then, a 2D crystal plasticity finite element method was adopted to simulate the local stress distribution under different applied loads and loading frequencies. Simulations showed that the maximum residual local stress was much smaller under ultrasonic frequency loading in contrast to that under conventional frequency at the same applied load. It was also revealed that the maximum local stress increases with the applied load under both loading frequencies. The accumulated plastic strain was adopted as a fatigue indicator parameter to characterize the frequency effect, which was several orders smaller than that obtained under conventional loading frequencies when the applied load was fixed. The increment of accumulated plastic strain and the load stress amplitude exhibited a linear relationship in the double logarithmic coordinate system, and an improved fatigue life prediction model was established.

## 1. Introduction

Many key mechanical components of modern industrial facilities are required to endure cyclic loads exceeding 107 cycles during their service lives, while catastrophic accidents caused by fatigue failure are frequently reported [1,2,3,4,5]. Thus, it is crucial to assess the fatigue properties of materials and components in the very-high-cycle fatigue (VHCF) regimes, i.e., beyond 107 cycles. A fatigue experiment is a time-consuming job, where a fatigue life of up to 108 cycles can take about 23 days under a conventional loading frequency of 50 Hz. Therefore, accelerating fatigue tests with higher frequencies were developed to reduce the fatigue experimental time cost, and ultrasonic fatigue tests with a loading frequency of 20 kHz have been widely adopted since 1980 in VHCF studies [6,7]. It only takes about 1.4 h to achieve a fatigue test with 108 cycles, which brings great convenience to fatigue assessments compared with conventional ones with a frequency between 10 and 100 Hz. Some standards about ultrasonic frequency testing methods have been developed in Japan for the rapid generation of high- and VHCF data [8,9].

However, controversies concerning the ultrasonic fatigue test have arisen due to the significant difference in loading frequencies since it was proposed, and many investigations have been conducted. Irreversible microstructure degradation, such as dislocation multiplication and micro-plasticity, can lead to damage accumulation and failure. Compared to the conventional one, about three orders of magnitude higher strain rates are presented in ultrasonic frequency loading, which may lead to a great difference in micro-plastic behavior and alternative damage accumulation mechanisms. Several reviews concerning the frequency effect are given by Mayer et al. [10], Jeddi et al. [11], and Hong et al. [12]. It is generally accepted that the frequency effect is obvious for body-centered cubic materials, especially for those with low strength, while it is not obvious for face-centered cubic and hexagonal-close-packed materials.

Zhao et al. [13] studied the influence of loading frequency on a high-carbon bearing steel (GCr15) tempered at four different temperatures and proposed that the different dislocation moving distances between different loading frequencies are the key factors influencing fatigue lives. Guennec et al. [14,15] investigated the fatigue properties of a low-carbon steel (JIS S15C) with five different loading frequencies ranging from 0.2 Hz to 20 kHz, which revealed that the micro-plastic behaviors are quite different from each other at different loading frequencies and have direct influences on fatigue lives. Schneider et al. [16] studied the influences of loading frequency on the fatigue behavior of 50CrMo4 steel with two different strength levels and aluminum alloy EN AW-5083, indicating that the frequency effect is dependent on the strain rate dependency of materials. Torabian et al. [17] carried out low frequency and ultrasonic fatigue testing for a DP600 dual-phase steel, suggesting that rate-dependent flow behaviors of body-centered cubic metals are the decisive factors for the obvious loading frequency effect. Fintová et al. [18] studied the fatigue performances of EA4T (25CrMo4) steel extracted from the real railway axle with two loading frequencies of 120 Hz and 20 kHz, and the results suggested the same fatigue crack initiation mechanism but higher fatigue lives at ultrasonic frequencies. Hu et al. [19] studied the fatigue performances of three specimens whose tensile strengths are all larger than 1600 MPa. All of them show a slight frequency effect at ultrasonic frequencies. Fu et al. [20] investigated the effects of load frequency on the fatigue crack propagation behavior of selected laser-melting-processed Inconel 718 alloy, suggesting that a lower loading frequency leads to a higher concentration fluctuation and a larger penetration depth of hydrogen ahead of the crack tip and thus further promotes the acceleration of the fatigue crack growth rate. For face-centered cubic materials such as stainless steel [21], aluminum alloy [22] and hexagonal-close-packed materials such as titanium alloy [23], the investigation results demonstrate no obvious or just slight differences existing in fatigue lives as low and ultrasonic loading frequencies are applied, respectively. However, as the influences of environmental factors are considered [24,25], the frequency effect is noticeable.

Although there have been many studies on the frequency effect, these investigations are mainly carried out with experiments, and the frequency effect is interpreted from the roles of strain rate, microstructure type, temperature rise, and material strength state. Due to these complex influencing factors, controversial results are sometimes reported. Thus, a theory or effective computational method that can quantitatively describe the influence of frequency is eagerly desired.

In recent years, the crystal plasticity finite element method (CPFEM) has been proven to be an important and effective approach to studying the fatigue properties of materials [26,27]. Depicting grains with various orientations, a representative volume element (RVE) with stochastic gliding systems is established, and the accumulated plastic strains under each loading cycle can be calculated numerically, which makes it very useful in fatigue life predictions. In an earlier stage, CPFEM was mainly adopted to predict the lives of low cycle fatigue (LCF) [28,29] and creep fatigue [30,31,32,33], and many life prediction methods have been proposed and developed based on fatigue indicator parameters (FIPs) gained through CPFE analysis. Later, the accumulated plastic strain is also calculated with improved CPFEM and taken as the most important FIP to predict fatigue lives in the high cycle fatigue (HCF) and VHCF regimes.

Zhu et al. [34] predicted the LCF and HCF life of AISI 4140 steel under different stress amplitude ratios. Jiang et al. [35] evaluated nine FIPs that are related to micro-porosity in nickel-based single crystal superalloys and chose the maximum value of the stress–strain concentration factor and the geometric mean of the resolved stress–strain concentration factor to predict failure mode and LCF/HCF lives. Li et al. [36] proposed a novel high-low cycle fatigue life prediction model for Inconel 718 superalloy based on accumulated energy dissipation derived by CPFEM, which indicated that the experimental data are within a scatter band of ±2 on life prediction. A RVE model with different sizes of defects was established by Cong et al. [37] to analyze the effect of local stress–strain on the HCF and VHCF lives of a weathering steel Q450NQR1, which indicated that grains with large Schmid factors have higher accumulated plastic strain, and inclusions are mainly responsible for the large scatter of fatigue life dispersion. Shen et al. [38] proposed a crystal plastic constitutive model incorporating a back stress model obtained from electron back-scatter diffraction experiments of aluminum alloy A7075 and improved the accuracy of life prediction methods significantly. By incorporating a modified Ohno-Wang constitutive model considering the evolution of back stress into a CPFE model, Li et al. [28] constructed an HCF/VHCF life prediction model for 7075-T6 aluminum alloy, while the error between the experiments and simulations was within 27.2%. Back-stress-based stored energy was also taken as an FIP to locate the fatigue crack sites at twin boundaries and predict the lives of Inconel718 superalloy [39]. Studies on the VHCF life prediction of materials fabricated by additive manufacturing techniques have also been carried out these years. RVEs with different defects and CPFE models were constructed by Qian et al. [40,41] to predict the VHCF properties of AlSi10Mg fabricated with selected laser melting techniques, where the influences of build directions, pores, and inclusions were analyzed in detail. Prithivirajan et al. [42] utilized six fatigue metrics to investigate the failure locations and fatigue life predictions of Inconel 718 fabricated via selected laser melting techniques, and the results are beneficial in assessing the scatter of experimental results and accelerating the qualification process. Although CPFEM has been proven to have many advantages, the requirement for computing resources is an undeniable challenge. The Material Knowledge System was adopted to evaluate the HCF performance of microstructure [43] and extended to model the characteristics of a material in the transition fatigue regime between LCF and HCF [44]. Other methods have also been used to reduce computing costs and ensure calculation accuracy in HCF and VHCF simulations. An acceleration strategy that replaces the short-period waveform with a long-period waveform in the same period is used to describe the whole loading process of VHCF [45]. Cheng et al. [46] proposed an acceleration strategy based on the cycle-jump approach to make CPFE simulation applicable for high-cycle fatigue. Goash et al. [47] accelerated CPFE simulation by using the wavelet transformation-induced multi-time scaling method until crack nucleation to investigate the influence of microstructure and load sensitivity on dwell fatigue behavior.

In this paper, the fatigue behaviors of three specimens with three different strength levels were investigated through ultrasonic loading frequency (*f* = 20 kHz) and conventional loading frequency (*f* = 52.5 Hz), whose fractography and fatigue strength were obtained and mutually compared. Then, CPFEM was introduced and adopted to reveal the different residual stress distribution under the same applied load but different loading frequencies, which suggested different accumulated plastic strain. The accumulated plastic strains were also calculated and compared with each other under different applied loads and loading frequencies for three different specimens, which was adopted to explain the frequency effect. Finally, an improved fatigue life prediction model was established based on the increment of accumulated plastic strain.

## 2. Materials and Methods

### 2.1. Materials and Specimens

**Figure 1:**
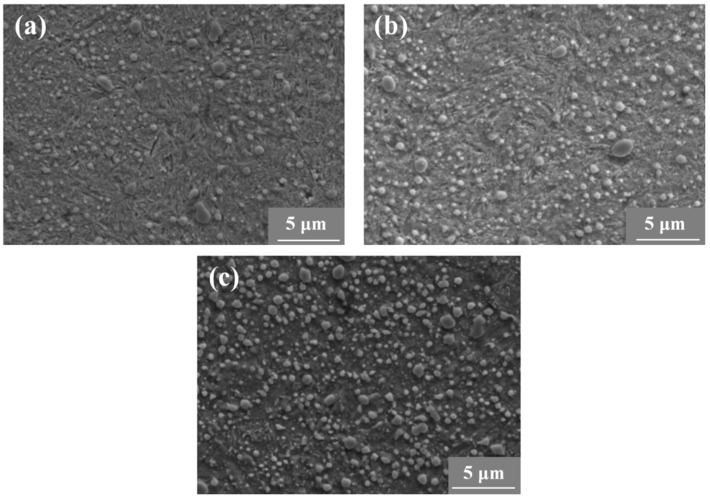
Scanning electron microscopy micrographs for two groups of specimens tempered at three different temperatures. (**a**) T.T. 300 °C, (**b**) T.T. 450 °C, and (**b**) T.T. 600 °C.

The material adopted in this study is a high-carbon chromium-bearing steel (GCr15), whose chemical composition is shown in Table 1. The specimens were heated at 845 °C in vacuum for 120 min, then oil-quenched and tempered in vacuum at 300 °C, 450 °C, and 600 °C for 150 min with furnace cooling, respectively. The microstructures of all three groups of specimens are shown in Figure 1. It is seen that the carbon element precipitates during the tempering process in the form of spheroidal carbides, and it is clear that both the size and number of spheroidal carbides increase with the tempering temperature. It is seen from Figure 1 that only a small amount of acicular-tempered martensite exists for the specimens at T.T. 300 °C (T.T. stands for tempering temperature hereafter). While for specimens T.T. 450 °C and T.T. 600 °C, the microstructures are troostite and tempered sorbite, respectively.

### 2.2. Experimental Methods

**Figure 2:**
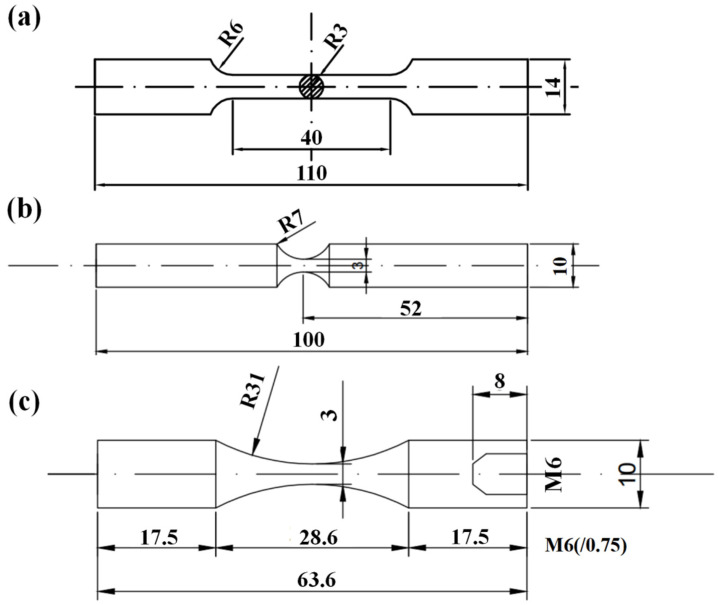
Geometrical dimensions of specimens used for experiments (dimensions in mm). (**a**) Uniaxial tensile experiment, (**b**) conventional fatigue tests, and (**c**) ultrasonic fatigue tests.

The geometries of the specimens for tensile and fatigue tests are shown in Figure 2. The surface of the specimens was polished by grade 600, 1000, 1500, and 2000 abrasive papers, successively. Cylindrical specimens with a diameter of 6 mm in gauged sections were used for the evaluation of the mechanical properties of the three groups of materials. The experiment was performed on an MTS 810 testing machine (MTS Systems Corporation, Eden Prairie, MN, USA), and the applied strain rate is 10^−4^ s^−1^. The conventional frequency fatigue experiments were carried out on a rotating bending machine at room temperature with a loading frequency of *f* = 52.5 Hz and the stress ratio *R* = −1. Sinusoidal waveform mode with a frequency of *f* = 20 kHz and the stress ratio *R* = −1 was used for ultrasonic fatigue tests.

### 2.3. Crystal Plasticity Theory

Within the framework of continuum mechanics [48,49], the total deformation gradient F can be decomposed into elastic part $F_{e}$ and plastic part $F_{p}$, which can be expressed as:

(1) $$F=F_{e}\cdot F_{p}$$

The elastic part $F_{e}$ associates with rigid body rotation and elastic stretching of the crystal lattice, and the plastic part $F_{p}$ origins from lattice slip.

The velocity gradient L in the current state can be calculated by the elastic velocity gradient $L_{e}$ and the plastic velocity gradient $L_{p}$:

(2) $$L=\dot{F}\cdot F^{-1}=L_{e}+L_{p}$$

L can also be decomposed into the symmetric rate of stretching tensor D and the antisymmetric spin tensor Ω. Both of them can be further decomposed into elastic and plastic parts, i.e.,:

(3) L=D+Ω

(4) $$D=D_{e}+D_{p}$$

(5) $$\Omega =\Omega_{e}+\Omega_{p}$$

In that case, $L_{e}$ and $L_{p}$ can be calculated by $F_{e}$ and $F_{p}$ respectively, that is:

(6) $$L_{e}=D_{e}+\Omega_{e}={\dot{F}}_{e}\cdot {F_{e}}^{-1}$$

(7) $$L_{p}=D_{p}+\Omega_{p}={\dot{F}}_{p}\cdot {F_{p}}^{-1}=\sum_{\alpha =1}^{N}{\dot{\gamma }}^{\alpha }s^{*\alpha }m^{*\alpha }$$

where N is the number of slip systems; ${\dot{\gamma }}^{\alpha }$ is the slip rate for the αth slip system; $s^{*\alpha }$ and $m^{*\alpha }$ are the vectors in the slip direction and normal to the slip plane of slip system in the deformed configuration, respectively, which can be calculated using:

(8) $$s^{*\alpha }=F_{e}\cdot s^{\alpha }$$

and

(9) $$m^{*\alpha }=m^{\alpha }\cdot {F_{e}}^{-1}$$

where $s^{\alpha }$ and $m^{\alpha }$ are respectively the unit vector in the slip direction and normal to the slip plane of slip system in the reference configuration.

Based on the Schmid law, Hutchinson [50] proposed a phenomenological rate-dependent constitutive model:

(10) $${\dot{\gamma }}^{\alpha }={\dot{\gamma }}_{0}{\left(\frac{\tau^{\alpha }}{g^{\alpha }}\right)}^{n}\mathrm{sgn}\left(\tau^{\alpha }\right)$$

where ${\dot{\gamma }}^{\alpha }$ is determined by resolved shear stress $\tau^{\alpha }$ and slip resistance $g^{\alpha }$; ${\dot{\gamma }}_{0}$ is the reference strain rate; n is the rate sensitivity exponent.

The hardening behavior of a slip system α is characterized by a slip system β, which can be expressed as:

(11) $${\dot{g}}^{\alpha }=\sum_{\beta =1}^{N}h_{\alpha \beta }\left|{\dot{\gamma }}^{\beta }\right|$$

According to the work of Pierce and Asaro [51], the latent hardening modulus $h_{\alpha \beta }$ and self-hardening modulus $h_{\alpha \alpha }$ in this work is given by:

(12) $$h_{\alpha \beta }=qh(\gamma )+(1-q)h(\gamma )\delta_{\alpha \beta }$$

and

(13) $$h_{\alpha \alpha }=h(\gamma )=h_{0}{\mathrm{sech}}^{2}\left|\frac{h_{0}\gamma }{\tau_{s}-\tau_{0}}\right|$$

where

(14) $$\gamma =\sum_{\alpha =1}^{N}\int_{0}^{t}\left|{\dot{\gamma }}^{\alpha }\right|\mathrm{dt}$$

Here, the constant *q* is 1.0 when the slip systems α and β are coplanar, and 1.4 otherwise. $h_{0}$, $\tau_{0}$ and $\tau_{s}$ are material constants, which represent initial hardening modulus, yield stress and the saturated flow stress (or the break-through stress where large plastic flow initiates), respectively. γ is Taylor cumulative shear strain on all slip systems.

Plastic strain and local stress are important factors in predicting crack initiation and fatigue lives. Accumulated plastic strain ($P_{\mathrm{ac}}$) represents the cumulative slip deformation caused by shear stress in all slip systems, which can be obtained by the double dot product of plastic strain gradients, that is:

(15) $$P_{\mathrm{ac}}=\int \sqrt{\frac{2}{3}L_{p}:L_{p}}\mathrm{dt}$$

## 3. Crystal Plasticity Finite Element Simulation

### 3.1. Finite Element Modeling

To evaluate the mechanical and VHCF properties of GCr-15, the simulations were conducted using the software ABAQUS (v.6.10) and a user-defined material subroutine (UMAT) program based on crystal plasticity theory introduced in Section 3.1.

**Figure 3:**
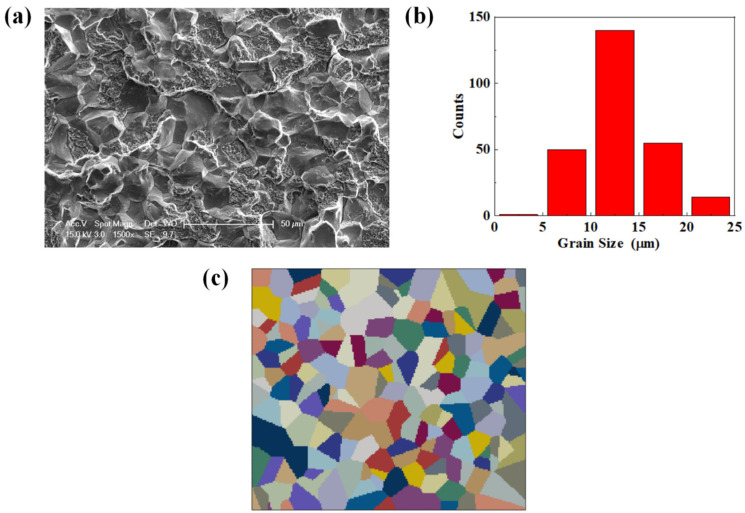
The distribution of prior austenite for GCr15 steel. (**a**) Typical mixed intergranular and transgranular fractography obtained from the fracture surface of specimen T.T. 300 °C. (**b**) the statistical distribution of grain sizes. (**c**) The 2D RVE established according to the distribution of grain sizes.

A typical mixed intergranular and transgranular fractography for specimens T.T. 300 °C is observed and presented in Figure 3a, and obvious prior austenite grains are observed. The statistical distribution is shown in Figure 3b, where a total of 821 grains are counted and the average grain size is about 13.8 µm. Based on the statistical results, a 2D finite element RVE with 200 grains is generated in the Voronoi method, as shown in Figure 3c, of which the height is 185 μm and the width is 210 μm. The RVE is meshed using four-node bilinear plane stress quadrilateral elements, and the total number of elements is about 38,000.

**Figure 4:**
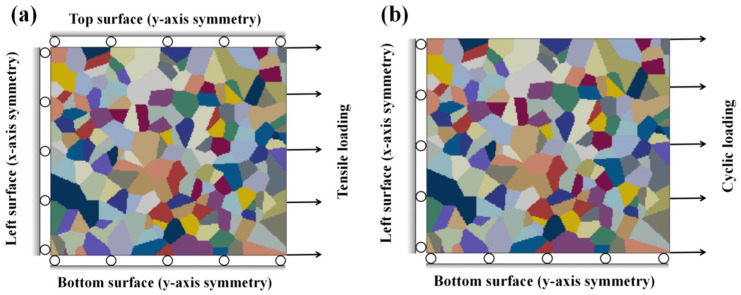
Boundary conditions for (**a**) tensile loading and (**b**) cyclic loading.

In the case of tensile loading, the boundary condition is set as shown in Figure 4a. The top and bottom surfaces are respectively subjected to a y-axis symmetry boundary condition, and the left surface is subjected to an x-axis symmetry boundary condition. In the case of fatigue loading, the y-axis symmetry boundary is only applied to the bottom surface, and the right surface is set to uniaxial tensile or cyclic loading along the x-axis, as shown in Figure 4b. Sine waves with different amplitudes and frequencies are adopted in the fatigue loading simulations.

### 3.2. Model Parameter Calibration

**Figure 5:**
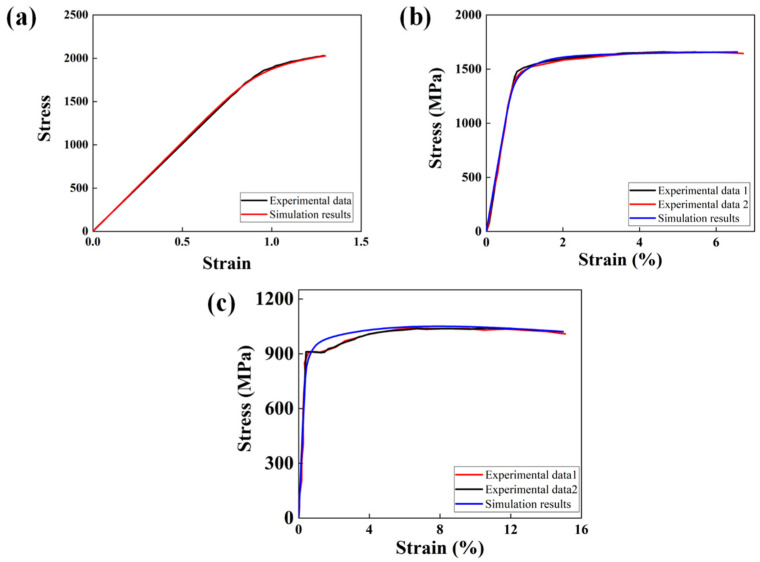
Stress–strain evolution of GCr-15 specimens under uniaxial tensile loading: (**a**) T.T. 300 °C; (**b**) T.T. 450 °C; (**c**) T.T. 600 °C.

The model parameters necessary for CPFE simulations can be obtained from the tensile experiments and the uniaxial tensile curves shown in Figure 5. The yield stresses for the specimens tempered at 300 °C, 450 °C, and 600 °C are 2000, 1537, and 909 MPa, respectively. Based on the CPFEM stated in Section 2.3, the tensile behaviors of three materials with different tempering temperatures (T.T. 300 °C, T.T. 450 °C, and T.T. 600 °C) were simulated to calibrate the model parameters. There are three slip systems for body-centered cubic metal crystals, which are {110}<111>, {112}<111>, and {123}<111>. Considering experiments were conducted at room temperature, only two slip systems, {110}<111> and {112}<111>, were implemented in the models of this study because the slip system of {123}<111> only activates at high temperatures [52,53].

The simulation results are shown in Figure 5, while the parameters obtained from simulations are listed in Table 2. Figure 5 illustrates that the parameters of the constitutive model were selected appropriately to repeat the curves from the experiments.

## 4. Results and Discussion

### 4.1. Fatigue Experimental Results

**Figure 6:**
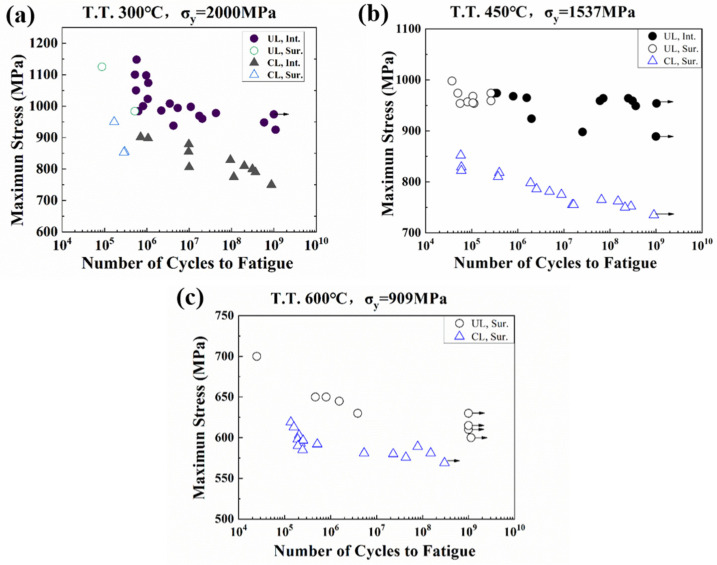
S–N curves of three groups of specimens under conventional frequency (CL) and ultrasonic frequency loading (UL): (**a**) T.T. 300 °C; (**b**) T.T. 450 °C; (**c**) T.T. 600 °C. σy denotes yield strength. Int. and Sur. refer to interior crack initiation and surface crack initiation, respectively [13].

Figure 6a presents the S-N data for the three groups of specimens obtained from conventional frequency fatigue loading (*f* = 52.5 Hz) and ultrasonic frequency fatigue loading (*f* = 20 kHz), where the circle symbols “○” represent fatigue data of conventional frequency and the triangle symbols “△” represent fatigue data of ultrasonic frequency. The data with an arrow “→” indicate unbroken specimens. It is seen that some specimens have fatigue lives beyond 10^9^. It is also evident that the S-N curves shift upward under ultrasonic frequency loading, suggesting that the fatigue lives are much larger at a higher frequency loading under the same applied stress amplitude.

**Figure 7:**
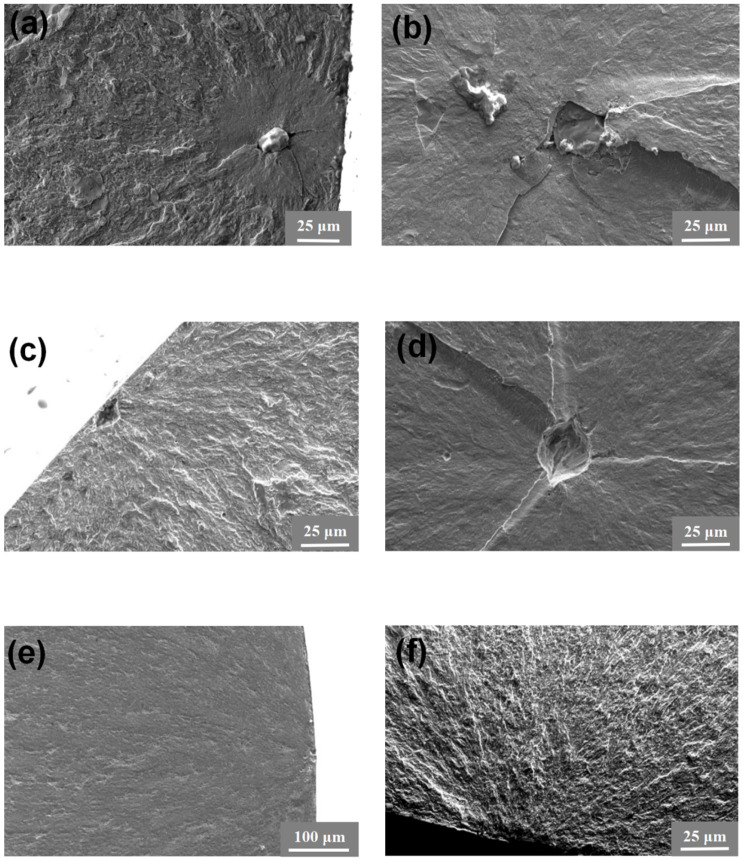
Typical morphologies of fracture surface for different specimens. (**a**) T.T. 300 °C, f = 52.5 Hz, stress amplitude *σ* = 879 MPa, number of cycles to fatigue *N_f_ =* 9.807 × 10^6^; (**b**) T.T. 300 °C, *f* = 20 kHz, σ = 938 MPa, *N_f_* = 4.254 × 10^6^; (**c**) T.T. 450 °C, *f* = 52.5 Hz, σ = 765 MPa, *N_f_* = 6.542 × 10^7^; (**d**) T.T. 450 °C, *f* = 20 kHz, σ = 874 MPa, *N_f_* = 2.116 × 10^8^; (**e**) T.T. 600 °C, *f* = 52.5 Hz, σ = 580 MPa, *N_f_* = 2.218 × 10^7^; (**f**) T.T. 600 °C, *f* = 20 kHz, σ = 650 MPa, *N_f_* = 8 × 10^5^.

The typical morphologies of the fractured surfaces of different specimens are displayed in Figure 7, where both internal crack initiation and surface crack initiation were observed. Internal crack initiation only occurs for specimens with the highest yield strength, and it is seen that fracture tends to initiate from the surface at high stress amplitudes and subsurface defects at low stress amplitudes.

### 4.2. Local Residual Stress Distribution Calculated by CPFEM

Similar to experimental conditions, two different frequencies were adopted: 20 kHz for ultrasonic fatigue tests and 52.5 Hz for conventional fatigue tests. For each loading frequency, 700, 800, 900, 1000, and 1100 MPa were respectively performed as stress amplitudes to specimens tempered at 300 and 450 °C, while 550, 600, 650, and 700 MPa were applied to specimens tempered at 600 °C. A sine wave was used, and the loading ratio was R = −1.

**Figure 8:**
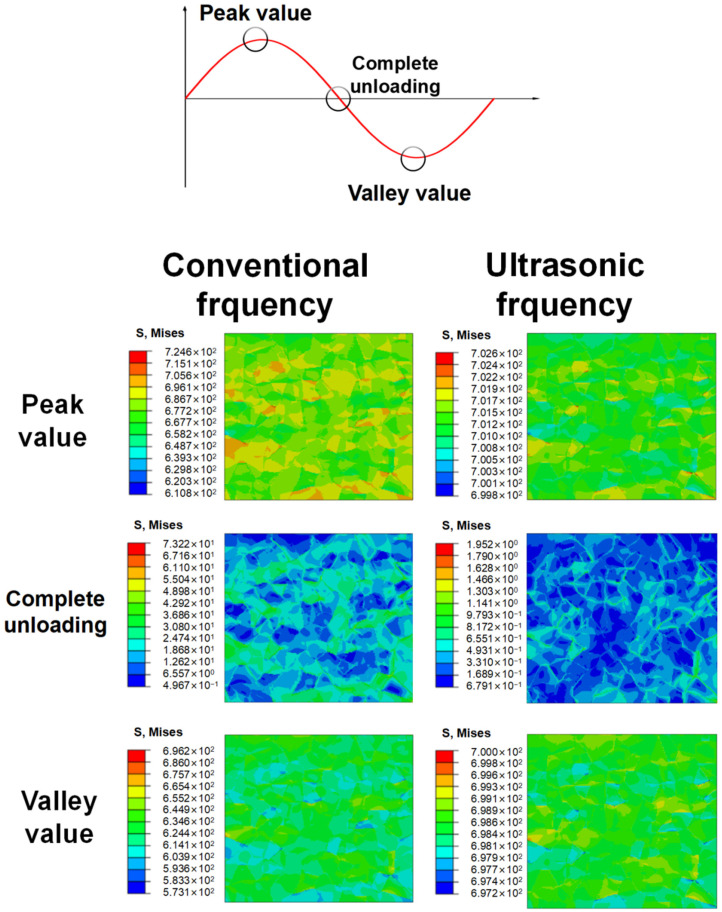
The stress fields of RVEs at three loading stages obtained through CPFEM for specimens T.T. 600 °C loaded at stress amplitude of 650 MPa after 100 cycles of loading.

Due to the restriction on computation abilities, only 100 cycles of iterations are carried out. The cloud map interval is in logarithmic form. The distribution of stress during the 100th loading cycle, corresponding to the applied load at the time of peak value, complete unloading, and valley value, is shown in Figure 8. When completely unloaded, residual stress concentration is more obvious under conventional frequency loading. Not only that, it is observed that the grains with larger residual stress bear higher stress at the time of peak value but lower stress at valley value during a loading cycle, which is attributed to the tensile and compressive asymmetry caused by grain orientation. Such a stress difference in the whole loading cycle may cause inconsistent deformation between grains and thus lead to more fatigue damage. When a specimen is loaded under ultrasonic frequency, the distribution of stress is much more uniform than that under conventional frequency. The stress concentration only occurs at the junction of more than two grains. Moreover, when a specimen is completely unloaded, the residual stress concentrates along grain boundaries rather than existing within the grains as it is loaded under conventional frequency.

**Figure 9:**
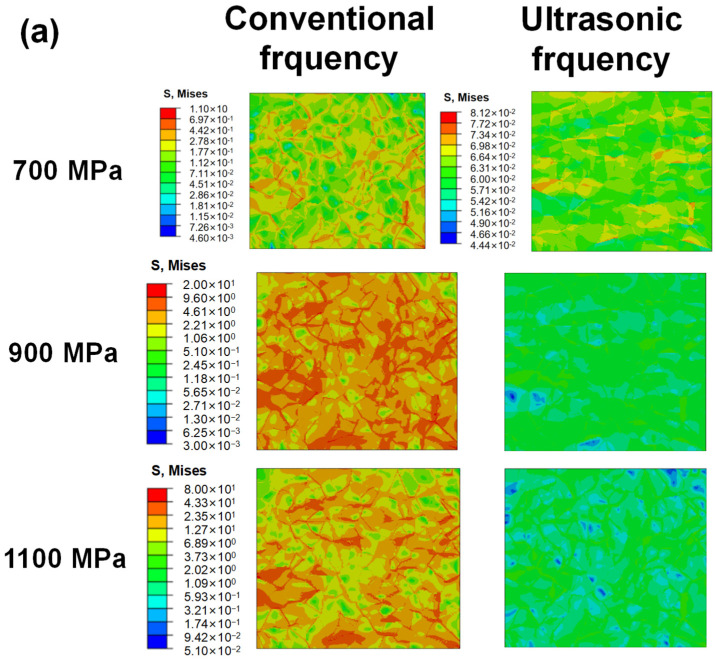

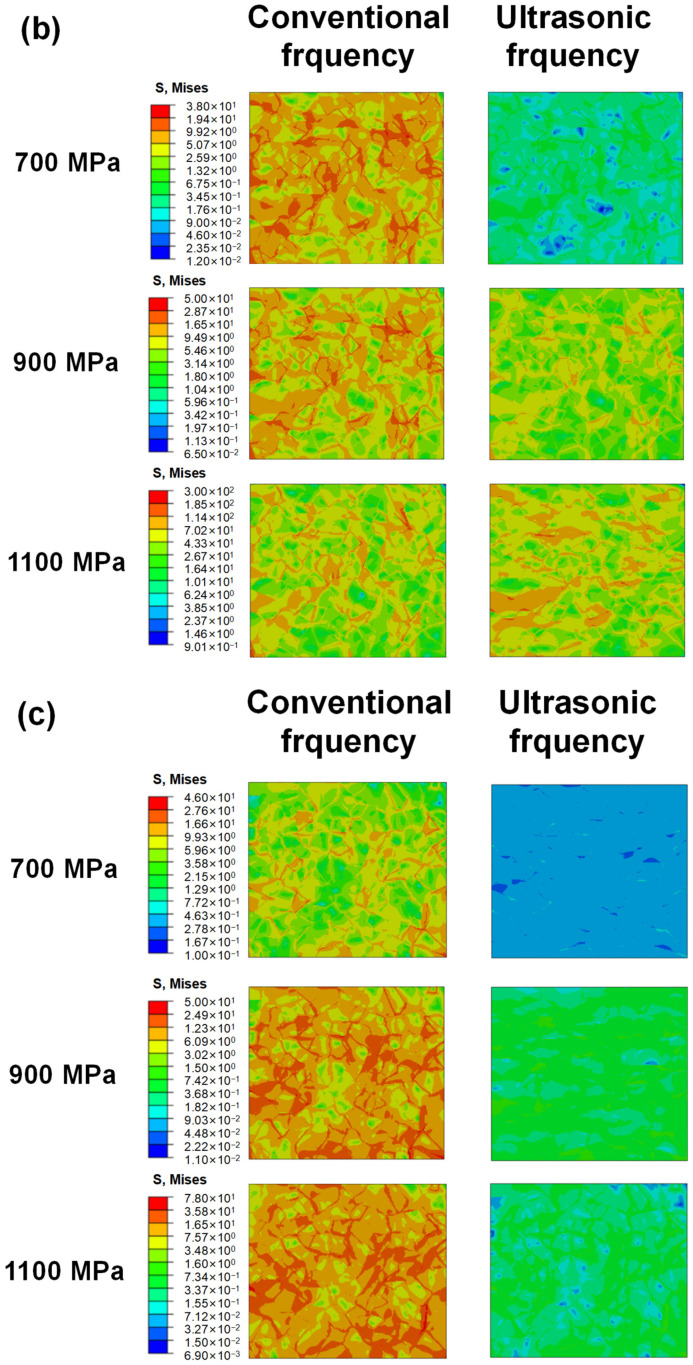
The local stress distribution obtained by RVEs through CPFEM for different specimens at different frequencies and stress amplitudes. (**a**) T.T. 300 °C. (**b**) T.T. 450 °C. (**c**) T.T. 600 °C.

Figure 9 shows the local residual stress distribution through CPFEM for different specimens at different loading frequencies and stress amplitudes. The results were captured after the 100th cycle. It is seen that, for cases loaded at ultrasonic frequency and low stress levels, the local stress distribution is relatively uniform (Figure 9a 700 MPa, 900 MPa; Figure 9c 550 MPa, 600 MPa). In other cases, the stress concentration occurs along the boundaries of grains, especially at the junction of more than two grains. The difference in orientation among adjacent grains is considered the main cause leading to the phenomenon. It is shown that the local residual stress is much lower when loaded under the same applied load but at ultrasonic loading frequencies, suggesting smaller fatigue damage under ultrasonic frequency loading.

### 4.3. The Accumulated Plastic Strain

**Figure 10:**
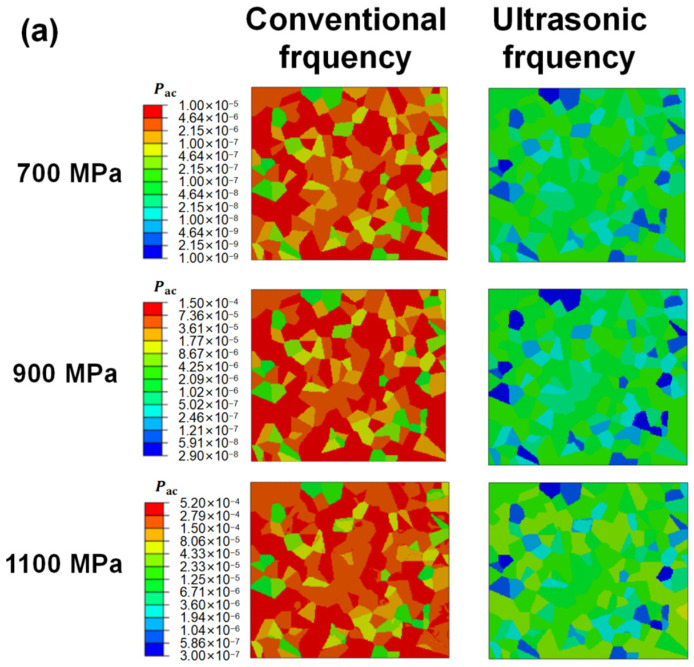

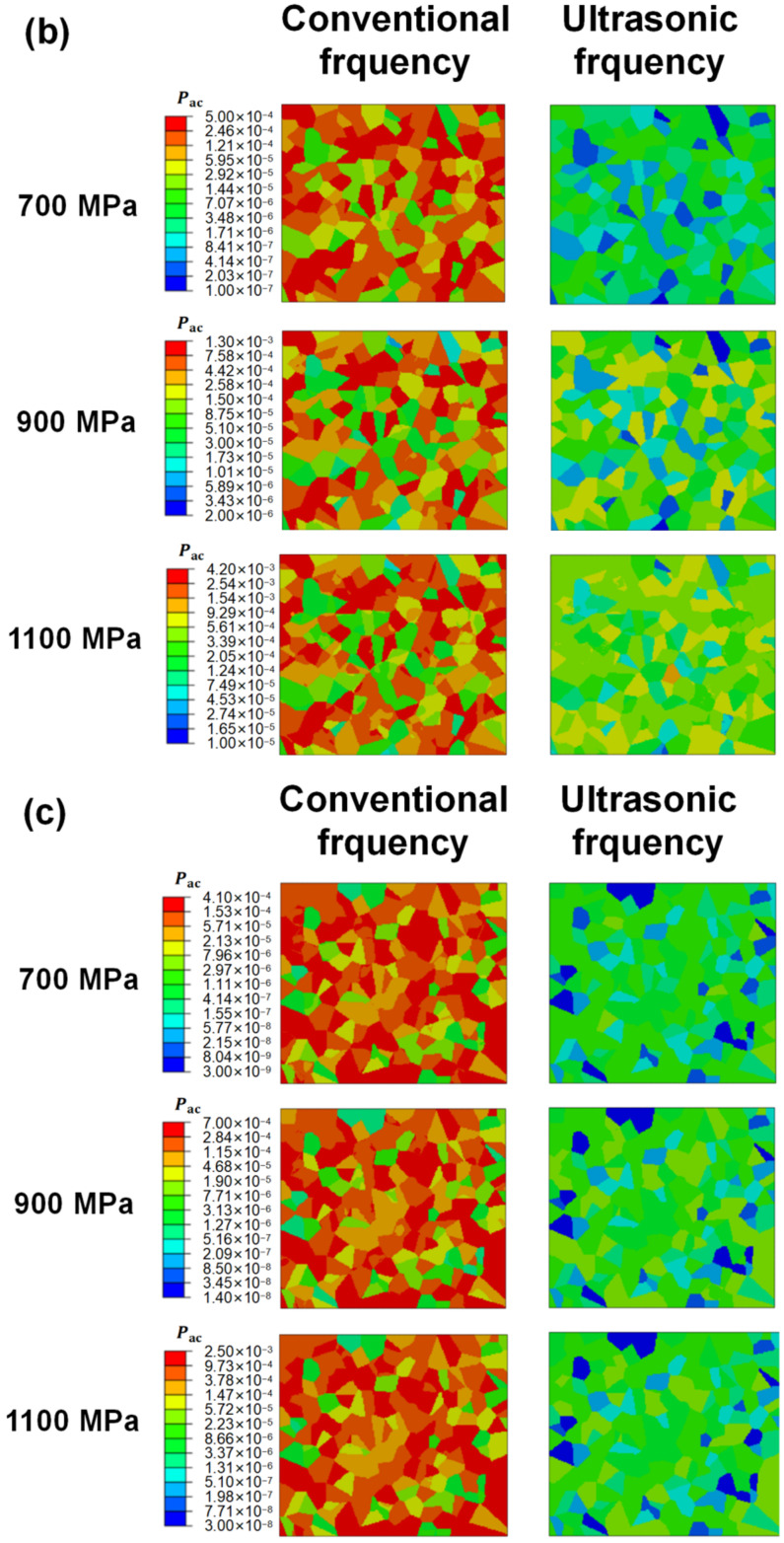
The distribution of accumulated plastic strain obtained by RVEs through CPFEM for different specimens at different frequencies and stress amplitude. (**a**) T.T. 300 °C. (**b**) T.T. 450 °C. (**c**) T.T. 600 °C.

Figure 10 shows the accumulated plastic strain fields in CPFEM models of T.T. 300 °C, 450 °C, and 600 °C under different applied loads, respectively. It is clearly seen that plastic strain exists even as applied loads are lower than yield stress due to the inhomogeneous deformation of grains, but the accumulated plastic strain (Pac) is uniformly distributed within most of the grains. During the simulation process, it is also observed that the accumulation of plastic strain starts around the boundaries and then develops to the interior of grains during the cyclic loading process. A few grains also show the phenomenon that the deformation is relatively uneven in a single grain (Figure 10a, 1100 MPa, 52.5 Hz; Figure 10b, 52.5 Hz; Figure 10c, 52.5 Hz) after 100 cycles of loading. For the specimens T.T. 600 °C loaded under 700 MPa, the locations of grains with a higher accumulated plastic strain are just the same as those bearing larger stress, as the applied load is at peak and valley values. The stress difference between adjacent grains and plastic strain accumulation is caused by the misorientation of grains. Because the fracture of specimens generally occurs under tensile load, the grains bearing a higher tensile stress are possible locations for crack initiation, especially as a specimen is loaded at conventional frequency.

Due to the huge difference in accumulated plastic strain under different applied loads and loading frequencies, different scale colors are adopted in each subfigure. It is clearly noticed that the maximum accumulated plastic strain increases with the amplitude of applied load at the same loading frequency. Under the same applied load but a different loading frequency, the maximum accumulated plastic strain is much smaller under ultrasonic frequency loading, which suggests much higher fatigue lives under the higher loading frequency reported in the previous fatigue experiments. Moreover, it is also noticed that under the same applied load and loading frequency, the accumulated plastic strain increases with the decrease in material strength, suggesting that the fatigue life will decrease with material strength and it is in accordance with the experimental results. Thus, the above phrasings indicate that the CPFE analysis can explain the fatigue experimental results qualitatively.

**Figure 11:**
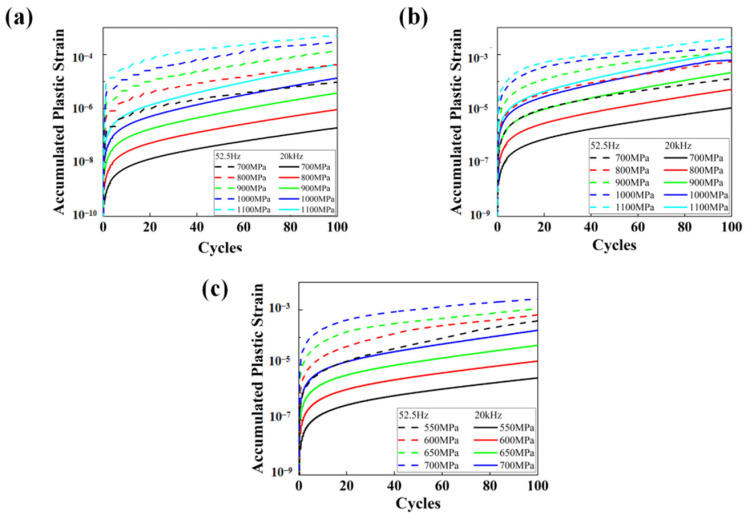
The variations of Pac for CPFEM models at different frequencies and load stress amplitudes. (**a**) T.T. 300 °C. (**b**) T.T. 450 °C. (**c**) T.T. 600 °C.

Figure 11 displays the variations of accumulated plastic strain $P_{\mathrm{ac}}$ for RVEs of specimens T.T. 300 °C, 450 °C, and 600 °C at different loading frequencies and applied stress amplitudes. The plastic strain accumulation process can be divided into two stages in double logarithmic coordinates. In the primary stage, the plastic strain accumulation starts at a very fast rate, and the accumulation rate decreases gradually. While in the secondary period, the growth of $P_{\mathrm{ac}}$ is slow and steady, and the increment of $P_{\mathrm{ac}}$ ($\Delta P_{\mathrm{ac}}$) remains almost constant. The phenomena well interpret the fatigue experimental results displayed in Figure 6. The specimens loaded at ultrasonic frequency need more cycles to cause failure, which means the damage caused by plastic strain in each cycle is less than that loaded at conventional frequency. It can be concluded that the accumulated plastic strain under ultrasonic loading is several orders smaller than that derived under conventional frequency loading. The much lower $\Delta P_{\mathrm{ac}}$ under ultrasonic frequency loading could explain the higher fatigue lives quantitatively.

### 4.4. Fatigue Life Prediction Model

Sun [39] compared three different life prediction models and found the model that considered grain size and $\Delta P_{\mathrm{ac}}$ is relatively reliable. Based on that, the predicted number of cycles to fatigue (*N_p_*) can be quantified by the influence of frequency and material strength. It is:

(16) $$N_{p}=\frac{\xi }{d_{\mathrm{gr}}}\frac{1}{{\left(\Delta P_{ac}\right)}^{\zeta }}$$

where *dgr* is a constant that reflects grain size. In this study, it is 13.8 × 10^−3^. *ξ* is a fitting constant. *ζ* is a constant that reflects the sensitivity of materials with different strengths to frequency.

**Figure 12:**
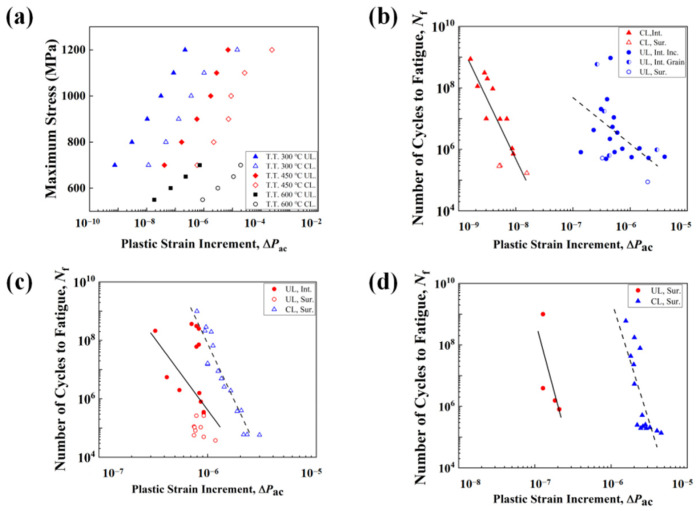
(**a**) The relationship between the load stress amplitude and the increment of accumulated plastic strain ($\Delta P_{\mathrm{ac}}$). The fitting results between the number of cycles (*N_f_*) to fatigue and $\Delta P_{\mathrm{ac}}$ for specimens (**b**) T.T. 300, (**c**) T.T. 450 and (**d**) T.T. 600 °C.

**Figure 13:**
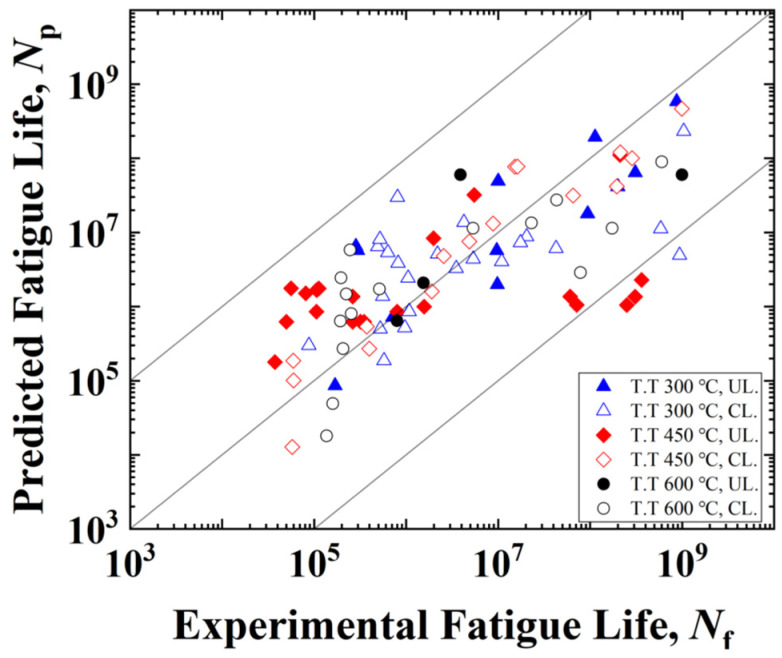
A comparison between the predicted fatigue life and experimental results.

Figure 12a shows that $\Delta P_{\mathrm{ac}}$ is linearly related to maximum stress. Considering experimentally loading stresses are all within the range of that in CPFE simulations, the interpolated $\Delta P_{\mathrm{ac}}$ of each frequency and applied load can be obtained to predict the fatigue life. The prediction results based on Equation (16) are shown in Figure 12b–d. Except for the results of specimens T.T. 300 °C loaded at 52.5 Hz and T.T. 450 °C loaded at 20 kHz, the prediction model fits well. The fitting errors in T.T. 300 °C loaded at 52.5 Hz and T.T. 450 °C at 20 kHz can be attributed to the interior crack initiations from inclusions and big grains, which are not considered in this work. In Figure 13, the experimental fatigue life and the predicted fatigue life are compared with each other. Results showed that most of the fatigue life data was located within the two-scatter band with ±2 errors. The four data points out of the scatter band are from cases of T.T. 300 °C, 52.5 Hz, and T.T. 450 °C, 20 kHz, which can be explained by the fitting errors of S-$\Delta P_{\mathrm{ac}}$ curves. Even so, the results are conservative, so the predicted fatigue life based on Equation (16) and the linear relationship between stress and $\Delta P_{\mathrm{ac}}$ is satisfactory.

## 5. Conclusions

In this paper, the fatigue performance of a high-strength bearing steel tempered at three different temperatures was investigated with ultrasonic frequency loading (*f* = 20 kHz) and conventional frequency loading (*f* = 52.5 Hz). The crystal plasticity finite element method (CPFEM) was adopted to explain the frequency effect quantitatively. The main results are summarized as follows:

(1) The yield strength of the high-carbon chromium-bearing steel lifts from 909 to 2000 MPa as the tempering temperature reduces from 600 °C to 300 °C. The S-N curves shift upward under ultrasonic frequency loading, suggesting that the fatigue lives were much larger at higher frequency loading under the same load stress amplitude. This frequency effect is obvious for all three groups of specimens.
(2) The local residual stress distribution obtained under the same applied load but at different loading frequencies indicates that the maximum local stress is much lower at ultrasonic frequency loading. The maximum local stress increases with the applied loads under both loading frequencies.
(3) Accumulated plastic strain can be taken as the fatigue indicator parameter to explain the frequency effect. For all three groups of specimens, the accumulated plastic strain obtained under ultrasonic loading frequency is several orders smaller than that obtained under conventional loading frequency, as the applied load is fixed. This is the reason for much higher fatigue lives under ultrasonic loading.
(4) The increment of accumulated plastic strain and the load stress amplitude exhibited a linear relationship in the double logarithmic coordinate system. Based on that, an improved fatigue life prediction model was established. The predicted results and the experimental fatigue life are in good agreement.

For the purpose of obtaining a more accurate simulation of micro-deformation, two aspects should be considered in future work. First, the influence of inclusion and big grains should be considered within the RVE. Second, the temperature rise caused by energy dissipation and back stress should be considered into the constitutive model. The temperature rise can affect the activation of possible slip systems for body-centered cubic materials and back stress can cause ratcheting strain.

## Figures and Tables

**Table 1:** Chemical composition of the tested GCr15 steel samples (wt.%).

C	Cr	Mn	Si	P	S	Fe
1.01	1.45	0.35	0.28	0.015	0.01	Balance

**Table 2:** Crystal plasticity model parameters of the GCr15 steel tempered at different temperatures.

Tempering Temperature	Slip System	n	*h*_0_ (MPa)	*τ*_s_ (MPa)	*τ*_0_ (MPa)
300 °C	{110}<111>	10	390	1000	570
	{112}<111>	10	290	1000	640
450 °C	{110}<111>	10	200	900	400
	{112}<111>	10	150	910	400
600 °C	{110}<111>	10	300	330	270
	{112}<111>	10	250	340	280

## Data Availability

The data that support the findings of this study are available from the corresponding author upon reasonable request.
